# The “Black Straight-Line Sign” in the Putamen in Diffusion-Weighted Imaging: A Potential Diagnostic MRI Marker for Multiple System Atrophy

**DOI:** 10.3389/fneur.2022.890168

**Published:** 2022-05-19

**Authors:** Yiming Zheng, Xiwen Wang, Huajian Zhao, Yanyan Jiang, Ying Zhu, Jing Chen, Wei Sun, Zhaoxia Wang, Yunchuang Sun

**Affiliations:** ^1^Department of Neurology, Peking University First Hospital, Beijing, China; ^2^Beijing Key Laboratory of Neurovascular Disease Discovery, Beijing, China; ^3^Department of Neurology, Hebei Province Cangzhou Hospital of Integrated Traditional and Western Medicine, Hebei, China; ^4^Department of Neurology, University of Chinese Academy of Sciences Shenzhen Hospital (Guangming), Shenzhen, China; ^5^Department of Radiology, Peking University First Hospital, Beijing, China

**Keywords:** multiple system atrophy, diffusion-weighted imaging, MRI, putamen hypointensive signal, Parkinson's disease

## Abstract

**Background and Purpose:**

The diagnosis of multiple system atrophy (MSA) remains challenging in clinical practice. This study investigated the value of hypointense signals in the putamen (“black straight-line sign”) in diffusion-weighted imaging (DWI) of brain MRI for distinguishing (MSA) from Parkinson's disease (PD).

**Methods:**

We retrospectively enrolled 30 MSA patients, 30 PD patients, and 30 healthy controls who had undergone brain MRI between 2016 and 2020. Two readers independently assessed the signal intensity of the bilateral putamen on DWI. The putaminal hypointensity was scored using 4-point visual scales. Putaminal hypointensity and the presence of a “black straight-line sign” were statistically compared between MSA and PD or healthy controls.

**Results:**

The mean scores of putaminal hypointensity in DWI in the MSA group were significantly higher than in both the PD (*U* = 315.5, *P* = 0.034) and healthy control groups (*U* = 304.0, *P* = 0.022). Uni- or bilateral putaminal hypointensity in DWI with a score ≥2 was identified in 53.3% (16/30), 16.7% (5/30), and 13.3% (4/30) of MSA, PD, and healthy controls, respectively, with significant differences between MSA and PD (X^2^ = 8.864, *P* = 0.003) or healthy controls (X^2^ = 10.800, *P* = 0.001). Notably, the “black straight-line sign” of the putamen was observed in 16/30 (sensitivity 53.3%) patients with MSA, while it was absent in PD and healthy controls (specificity 100%). There were no significant differences for the presence of “black straight-line sign” in the MSA-P and MSA-C groups (X^2^ = 0.433, *P* = 0.510).

**Conclusion:**

The “black straight-line sign” of the putamen in DWI of head MRIs has the potential to serve as a diagnostic marker for distinguishing MSA from PD.

## Introduction

Multiple system atrophy (MSA) is a rare neurodegenerative disease with the pathological hallmark of oligodendrocyte inclusion bodies (GCI) composed of alpha-synuclein aggregation (1). Clinically, the main manifestations of MSA are autonomic dysfunction superimposed with motor disorders of varying extent, specifically either Parkinsonian type (MSA-P) with Parkinson's syndrome as the main manifestation and cerebellar type (MSA-C) with cerebellar ataxia as the main manifestation (1). The MSA diagnostic criteria were revised in 2008 by Gilman et al. (2); however, its diagnosis remained challenging for clinical neurologists. For example, when autonomic dysfunction is not obvious until the advanced stage or when it only manifests as isolated Parkinsonism or cerebellar ataxia (1, 3, 4). It is therefore difficult to distinguish MSA from other Parkinsonisms, such as idiopathic Parkinson's disease (PD), progressive supranuclear palsy, and progressive ataxia such as spinocerebellar ataxia (1, 4, 5). Despite being the focus of various studies, the biomarkers used for MSA diagnosis, such as detection of alpha-synuclein in serum or cerebrospinal fluid, still lack generalized application and their accuracy remains unconfirmed (6, 7). Imaging findings play an important role in the diagnosis of MSA. The classic “hot cross bun” sign, “hyperintense putaminal rim” sign, cerebellopontine atrophy, an abnormally high signal in the pontine peduncle, and other abnormalities that reflect neuronal cell death and gliosis on structural magnetic resonance images (MRI) are widely known, but they are not specific to MSA (8, 9). The application of functional MRI has a certain diagnostic value, but it is difficult to perform these complicated examinations and analyses in routine clinic conditions (10, 11). Recent research has revealed that a hypointense putaminal signal on susceptibility-weighted imaging (SWI) of MRI is of great significance in the diagnosis of MSA (12, 13). A signal hypointensity score over 2 [unilateral or bilateral, a score of 2 when the intensity was similar to the Vein of Galen and with a posterolateral gradient; and score of 3 when marked posterolateral to anteromedial hypointensity (13)] in the putaminal margin is specific to MSA. Currently, we also find that there are MSA patients who also have similar hypointense signals in the putamen on the diffusion-weighted imaging (DWI) sequences and the low signals at the edge of the putamen show a straight distribution—which we term the “black straight-line sign”. The characteristics of the “black straight-line sign” and its diagnostic value in MSA remains unknown. The current study explored the characteristics of the putaminal “black straight-line sign” and its differential diagnosis between MSA, PD, and normal controls.

## Materials and Methods

This study was approved by the ethics committee of Peking University First Hospital in accordance with the Declaration of Helsinki. Each participant or their legal guardians signed a written informed consent before participating in the study.

### Subjects and Patient Consents

This was a retrospective study undertaken in the department of neurology at the Peking University First Hospital. Thirty consecutive inpatients with MSA, 30 non-consecutive inpatients with PD, and 30 age-matched, normal, healthy controls were enrolled from 2016 to 2020. The MSA patients were diagnosed as “probable MSA” based on the second consensus clinical criteria (2). The MSA group included 19 MSA-P (predominant Parkinsonian features) and 11 MSA-C (predominant cerebellar features) patients. The PD patients were diagnosed using the Movement Disorder Society Criteria and all PD patients fulfilled with the clinically established PD (14). All MSA and PD patients were clinically assessed at the first visit and confirmed the diagnosis again during this study by an experienced neurologist (JC, YS, and ZW). All the healthy controls reported no major neurological or psychiatric diseases and none of the positive signs were detected during neurological examinations. The following demographic and clinical information of the MSA and PD subjects, including gender, age at evaluation, disease duration, and Hoehn and Yahr (H-Y) stages (15), were abstracted from medical records.

### MRI Protocol and Image Evaluation

All participants underwent a 1.5 or 3.0 Tesla MRI scanning (MAGNETOM Aera 1.5T Siemens Healthcare, Erlangen, Germany. Ingenia 3.0T, Philips Medical Systems, Netherlands). The parameters of DWI of the 1.5 T MR were as follows: repetition time (TR) = 7,280 ms; echo time (TE) = 81 ms; slice number = 20; slice thickness = 6mm slice gap = 0.9mm; flip angle = 180; field of view (FOV) = 240 × 240 mm2; voxel size = 1.3 × 1.3 × 6 mm. The parameters of DWI of the 3.0 T MR were as follows: repetition time (TR) = 4,000 ms; echo time (TE) = 72 ms; slice number = 20; slice thickness = 6 mm slice gap = 1 mm; flip angle = 90; field of view (FOV) = 230 × 230 mm2; voxel size = 1.44 × 1.44 × 6 mm; NSA = 1.

**Figure 1:**
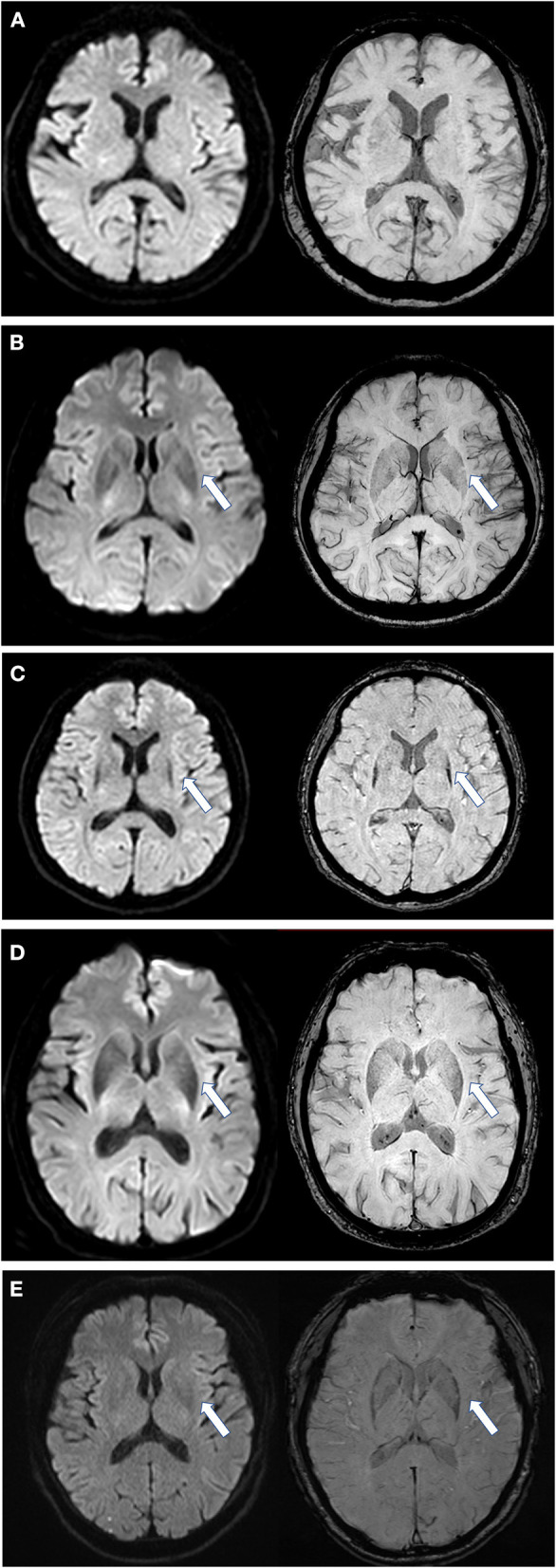
Scores of putaminal hypointensity in DWI and the corresponding SWI sequence. Left, DWI sequence; right, SWI sequence. **(A)** DWI and SWI score are all 0 as shown by an arrow; **(B)** DWI and SWI score are all equal to 1; **(C)** DWI and SWI scores are all 2 points, and in a straight distribution; **(D)** DWI and SWI scores are all 2, but with an arc shape and an unclear boundary; **(E)** from a PD patient, with a DWI score of 1 and SWI score of 2. DWI, diffusion-weighted imaging; SWI, susceptibility-weighted imaging. All illustrations are denoted as arrow.

**Figure 2:**
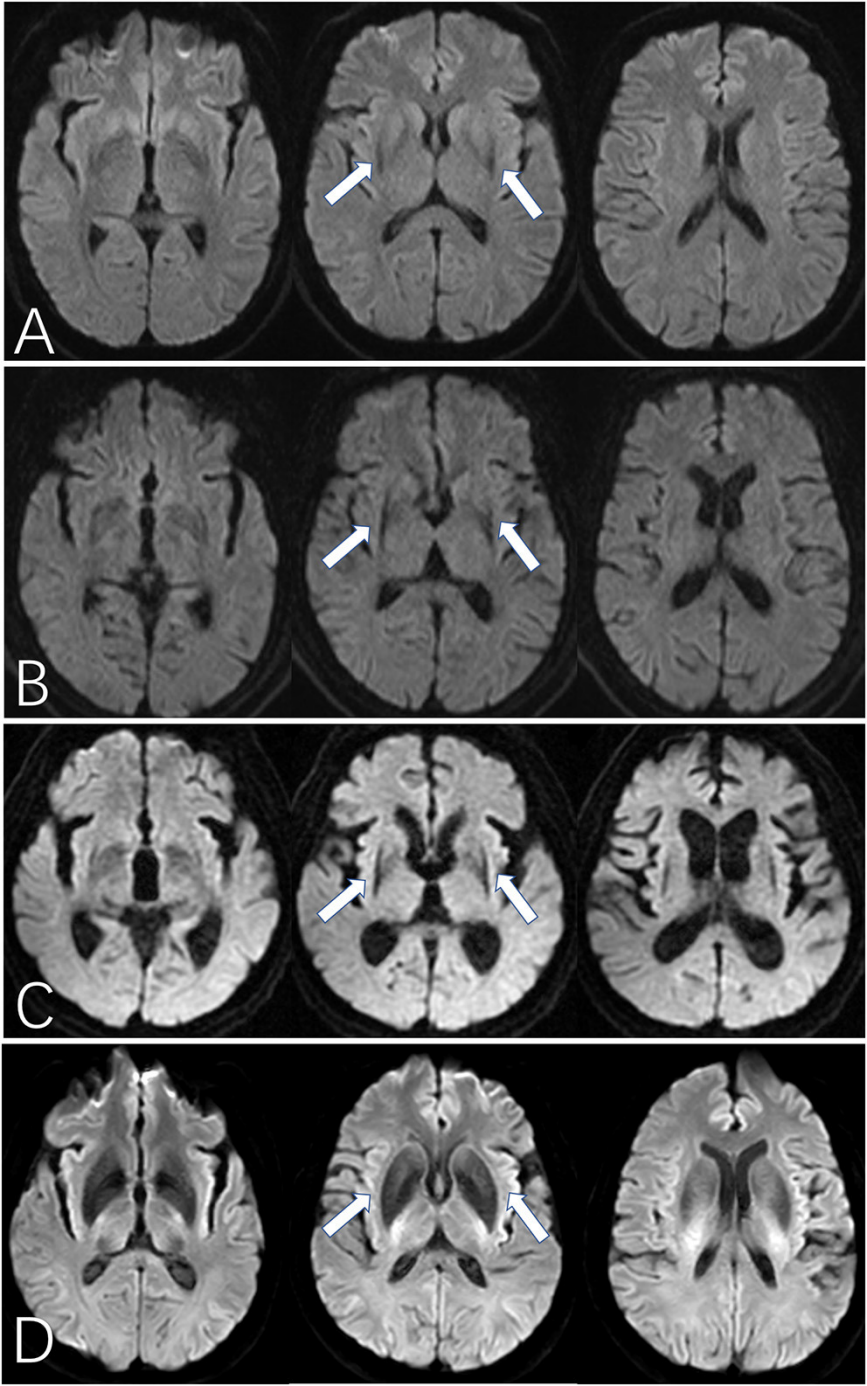
The “black straight-line sign” on DWI for MSA patients. **(A–C)** show the “black straight-line sign” from mild to obvious, showing a linear, abnormally low signal with a clear border in the putamen (arrow). The most obvious section is at the fornix. The “black straight-line sign” can be asymmetric **(B)** or symmetric **(C)**. **(D)** shows the hypointense putamen signal distributed in an arc in a healthy control subject. MSA, multiple system atrophy. All illustrations are denoted as arrow.

The signal intensity and the location of each putaminal abnormality on the DW images were evaluated separately by two readers with 14 and 8 years of neuroimaging MRI research experience (YS and MYZ) in a blind manner in which the demographic and disease information were concealed. If the score of the evaluation was inconsistent between the two readers in a given subject, the final grade for analysis was decided by a consensus between them. The evaluation process is detailed as follows:

First, we assessed the margin of the putamen on DW images, the evaluation of which was similar to SWI assessment (Figure 1) (12): Score of 0: the intensity was normal, no hypointense signal was observed; Score of 1: the intensity was similar to cerebrospinal fluid and without a posterolateral gradient; Score of 2: the intensity was similar to cerebrospinal fluid with a posterolateral gradient; Score of 3: the intensity was similar to cerebrospinal fluid with a marked gradient of posterolateral to anteromedial hypointensity. A hypointense signal score over 2 in the putamen margin was considered a criterion for an MSA diagnosis. If the scores of the two sides (right/left) differed, the higher score of the unilateral side and the mean score of both sides were calculated separately. The scores were graded previously to the imaging analysis. Second, we assessed the shape of the putaminal margin hypointensity in subjects with a score ≥2. If the low signal at the edge of the putamen showed a straight distribution (the “black straight-line sign”), it was considered a criterion for diagnosing MSA. If the low signal at the edge of the putamen was distributed in an arc, it was considered as a non-MSA abnormal signal (Figures 1, 2).

Third, other image parameters including SWI scores of putaminal hypointensity (12), hyperintense putaminal rim, hyperintensity of the pons (“hot cross bun” sign, including only cruciform hyperintensity), brain stem atrophy, and cerebellar atrophy were also evaluated by the two readers (16, 17).

### Statistical Analysis

Data were processed using SPSS 23.0 statistical software (SPSS Inc., Chicago, IL, USA). Continuous data were denoted as Mean ± SD, categorical data were denoted as Number (%). Shapiro-Wilk tests were used to assess the normality of continuous variables. A Kendall's tau-b grade correlation was used to evaluate inter-rater agreement on DWI. A Mann-Whitney U test was used to compare the grade of putaminal hypointensity on DWI between groups. Oneway analysis of variance (ANOVA), a chi-square test, and a Mann-Whitney U test were used to compare the differences for the clinical data and the “black straight-line sign” between groups. An independent sample *t*-test was used for age comparisons between two groups. Sensitivity (true positive/true positive + false negative) and specificity [true negative/(true negative + false positive)] parameters of the images were calculated. Results were considered significant at *p* < 0.05 (two-tailed).

## Results

### Comparison of Demographic and Disease Information

There were no significant between-group differences in age, gender, disease duration and 1.5 or 3.0 Tesla MRI among the MSA, PD, and healthy control groups. The MSA group had a significantly higher Hoehn and Yahr stage (*U* = 200.5, *p* < 0.001) than the PD group (Table 1).

**Table 1:** Demographic characteristics of the subject groups.

	**MSA (*n =* 30)**	**MSA-P (*n =* 19)**	**MSA-C (*n =* 11)**	**PD (*n =* 30)**	**HCs (*n =* 30)**	**MSA vs. PD**	**MSA vs. HCs**
Age (y)	62.2 ± 8.6	62.4 ± 9.3	61.9 ± 7.6	65.9 ± 6.9	60.0 ± 9.3	*t =*−1.854, *p =* 0.069	*t =* 0.965, *P =* 0.339
Gender (Male)	12 (40%)	9 (45%)	3 (27%)	14 (46.7%)	13 (43%)	*X*^2^ = 0.271, *P =* 0.602	*X*^2^ = 0.069, *P =* 0.793
Disease duration (y)	3.1 ± 2.2	3.1 ± 2.0	3.2 ± 2.6	3.2 ± 2.8	NA	U = 422.0, *p =* 0.676	NA
Hoehn and Yahr	3.2 ± 1.1	3.1 ± 1.1	3.6 ± 1.1	2.1 ± 0.9	NA	U = 200.5, p < 0.001	NA
3.0T MRI	14/30	10/19	4/11	12/30	16/30	*X*^2^ = 0.271, *P =* 0.602	*X*^2^ = 0.267 *P =* 0.606

*MSA, multiple system atrophy, PD, Parkinson's disease, HCs, healthy controls*.

### Comparison of Hypointense Putaminal Signals in DWI

The inter-rater reliability for putaminal hypointensity in DWI images was high (Kendall tau-b *R* = 0.849, *P* < 0.001). The putaminal hypointensity scores are summarized in Table 2. The scores (both mean and higher unilateral scores were significantly different among three groups with F = 4.007, *P* = 0.022 and F = 4.316, *P* = 0.016, respectively) of putaminal hypointensity in the MSA group were significantly higher than those in the PD and healthy control groups. There were no significant differences in putaminal hypointensity scores (both mean and higher unilateral scores) between the PD and healthy control groups. Uni- or bilateral putaminal hypointensity with a score ≥2 was significantly more common in MSA than in both PD and healthy controls and was identified in 53.3%(16/30), 16.7%(5/30), and 13.3%(4/30) of MSA, PD, and healthy controls, respectively. The mean and higher unilateral scores in 3.0T MRI were significantly higher than 1.5T MRI for the total population.(*U* = 581.000, *P* < 0.001 and *U* = 590.000, *P* < 0.001, respectively).

**Table 2:** DWI scores of putaminal hypointensity and other image parameters.

	**MSA** **(*n =* 30)**	**MSA-P** **(*n =* 19)**	**MSA-C** **(*n =* 11)**	**PD** **(*n =* 30)**	**HCs** **(*n =* 30)**	**MSA vs. PD**	**MSA vs. HCs**
**DWI scores of putaminal hyperintensity (Left; Right)**							
0 score	11; 11	6; 6	5; 5	16; 16	16; 16	-	
1 score	5; 4	2; 3	3; 1	9; 9	10; 11		
2 score	13; 15	10; 10	3;5	5; 5	3; 3		
3 score	1; 0	1; 0	0; 0	0; 0	1; 0		
Mean score	1.1 ± 0.9	1.3 ± 0.9	0.9 ± 0.9	0.6 ± 0.8	0.6 ± 0.7	U = 315.5, *P =* 0.034	U = 304.0, *P =* 0.022
Unilateral higher score	1.2 ± 1.0	1.3 ± 1.0	1.0 ± 1.0	0.6 ± 0.8	0.6 ± 0.8	U = 308.0, *P =* 0.024	U = 307.0, *P =* 0.024
≥2 score	16	11	5	5	4	*X*^2^ = 8.864, *P =* 0.003	*X*^2^ = 10.800, *P =* 0.001
**Shape of the hypointensity with a score** **≥2**						**Sensitivity**	**Specificity**
Straight	16	11	5	0	0	-	
Arc	0	0	0	5	4		
Black straight-line sign	16/30	11/19	5/11	0/30	0/30	53.3%	100%
**Other image parameters**							
SWI scores of putaminal hypointensity ≥2	4/6	3/5	1/1	5/21	0/19	66.7%	87.5%
“hot cross bun” sign	7/30	2/19	5/11	0/30	0/30	23.3%	100%
Hyperintense putaminal rim on T2	8/30	6/19	2/11	2/30	1/30	26.7%	95.0%
Brain stem atrophy	7/30	2/19	5/11	0/30	0/30	23.3%	100%
Cerebellar atrophy	15/30	7/19	8/11	1/30	0/30	50.0%	98.3%

*MSA, multiple system atrophy, PD, Parkinson's disease, HCs, healthy controls*.

When assessing the shape of the signal edge in cases with a score ≥2, we found that the rims of the low signal areas in the putamen of PD patients and healthy controls were always arcuate, while those of all the MSA patients were straight (Figure 2). The sensitivity of the “black straight-line sign” (uni- or bilateral putaminal hypointensity score ≥2 with a straight rim) in diagnosing MSA was 53.3% and the specificity was 100%. The diagnostic value of other image parameters including SWI feature of putaminal hypointensity, cerebellar atrophy, hyperintense putaminal rim, hyperintensity of the pons (“hot cross bun” sign) and brain stem atrophy were also displayed in (Table 2).

There were no significant differences in the proportion of cases with the “black straight-line sign” present between the MSA-P and MSA-C groups (*X*^2^ = 0.433, *P* = 0.510). No significant differences in age (*t* = −0.837, *p* = 0.410), disease duration (*U* = 84.500, *p* = 0.257), Hoehn and Yahr stage (U = 70.500, *p* = 0.085), or 1.5T/3.0T MRI (*X*^2^ = 1.265, *P* = 0.261) were observed between “black straight-line sign” positive and negative MSA patients. Except for SWI scores of putaminal hypointensity ≥2 (*X*^2^ = 6.000, *P* = 0.014), none of the other image parameters (i.e., hyperintense putaminal rim, hyperintensity of the pons, and brain stem atrophy) showed a significant difference between “black straight-line sign” positive and negative groups.

## Discussion

To our knowledge, this is the first study to assess the diagnostic value of hypointense putaminal signals in DWI between MSA, PD, and normal controls. Our results demonstrate that a score higher than 2, especially in the presence of a “black straight-line sign”, can differentiate MSA from PD and normal people. The “black straight-line sign” was specific to both MSA-P and MSA-C subgroups.

The hypointense signal in the posterior putamen was first noticed in the SWI sequence in MSA patients (12, 13, 18). Previous studies have found that this abnormal signal (unilateral or bilateral) with a score higher than 2 had high specificity in the diagnosis of MSA-P, although the sensitivity was relatively low (12, 13). We observed similar hypointense signal manifestations in DWI sequences among MSA patients in clinical practice, so we used a grading method similar to that used in SWI research in our study. Similar to the SWI studies, the current study found that the proportion of hypointense signals with a score >2 (unilateral or bilateral) in the posterior putamen in MSA was significantly higher than that in PD and normal healthy controls. However, we also found a high proportion of hypointense signals with scores over 2 in both PD and normal controls. After further assessment, we found that the morphological characteristics of this putaminal hypointensity in PD and normal controls were rather different from those in MSA. In MSA patients, the morphology of the hypointense signal was thin, straight, and bordered by the surrounding structure (i.e., black straight-line sign). On the contrary, in patients of PD and normal controls, the morphology of the hypointense signal was thick, with an arcuate shape consistent with the anatomical structure, and with a vague boundary with the surrounding normal structure. When the black straight sign was compared among the three groups, it was found that this sign only existed in patients with MSA, but not in PD and normal controls. Our results also show that although the sensitivity of the “black straight-line sign” is limited, its high specificity may be a novel imaging manifestation in the diagnosis of MSA.

We further graded the “black straight-line sign” into 3 different degrees. As shown in Figure 2, three different layers of the basal ganglia are presented on the axial image. A mild “black straight-line sign” is featured as a light abnormality appearing only in the middle plane. A moderate “black straight-line sign” is an obvious low signal degree appearing at the bottom and the middle planes, with a gradual trend but without pronounced posterolateral to anteromedial differences. An obvious “black straight-line sign” features a pronounced low-signal degree displayed at all three planes, with an obvious gradient from posterolateral to anteromedial shapes. Nonetheless, we did not find any association between the occurrence or severity of the “black straight-line sign” and disease duration or H-Y stages of MSA. Therefore, the emergence of this “black straight-line sign” should be viewed only as a diagnostic marker and not a grading marker of disease severity.

Previous research on hypointense posterior putaminal signals in SWI has mainly focused on the subgroups of MSA-P, while its characteristics in MSA-C were rarely investigated (12, 13, 18). In our study of the “black straight-line sign”, it appeared in a high proportion in both MSA-P and MSA-C, suggesting that this sign has limited significance in distinguishing the subtypes of MSA. This may reflect the fact that both MSA-P and MSA-C have similar neuropathological presentations despite the distributed scope of GCI being different in both subtypes (19). The “black straight-line sign” had comparable sensitivity and specificity in the diagnosis of MSA, which was comparable to the SWI feature of putaminal hypointensity ≥2 (with a sensitivity of 66.7%, and a specificity 87.5%) and cerebellar atrophy (with a sensitivity of 50.0%, and a specificity 98.3%) but better than the other image parameters, including the hyperintense putaminal rim (with a sensitivity of 26.7%, and a specificity 95.0%) and hyperintensity of the pons (“hot cross bun” sign) (with a sensitivity of 23.3% and a specificity 100%) in T2-weighted images, and brain stem atrophy (with a sensitivity of 23.3%, and a specificity of 100%). However, the fact that a hypointense posterior putaminal signal in SWI had a high false positivity (5/21,about 24%) in Parkinson's disease may limit its application. The combination of the “black straight-line sign” and posterior putaminal hypointensity in SWI may further improve the accuracy of disease diagnosis.

The reason for the appearance of the “black straight-line sign” in DWI remains unknown. Pathological results indicated that the neuronal cell loss, gliosis, and ferritin and Fe (3+) was predominantly located in the posterolateral part of the putamen (20–22). Because SWI sequences are highly sensitive to the paramagnetic effects of iron deposition in the putamen, we speculate that the causality is similar to that of the hypointense signal in the posterior putamen in SWI. Since the DWI sequence is also imaged based on the T2 sequence, we suspect that the appearance of the “black straight-line signal” also reflects the deposition of ferrous or iron ions. In addition, neuropathological and SWI image examinations showed obvious putamen atrophy in MSA (22, 23), which may account for its characteristic shape in DWI (i.e., clearly enclosed by the surrounding structures). More research is needed to explore the reason for the “black straight-line sign” on DWI.

Both 1.5 and 3.0 Tesla MRI scannings were used in this study. Previous studies demonstrated that as the field strength increased the occurrence of hypointensity at the dorsolateral putaminal margin increased in MSA (24). In this study, we also found that subjects that in 3.0 Tesla group had higher scores of hypointensity in the margin of the putamen on DW images. However, the occurrence of “black straight-line sign” in DWI showed no different between 1.5 and 3.0 Tesla MRI groups. Our study has several limitations: firstly, few patients underwent DWI and SWI imaging simultaneously, which may lead to limited accuracy of sensitivity and specificity of posterior putamen low signal sign in SWI. So more research is needed to compare the diagnostic value of the “black straight-line sign” and posterior putamen low signal sign in SWI. Secondly, our study did not include patients with other types of Parkinsonism, including progressive supranuclear palsy, dementia with Lewy body disease which were also difficult to differentiate from MSA in the early stages of the disease. Thirdly, neurologists did not blind to MRI data when establishing the diagnosis of these patients. Although they did not refer to DWI characteristics in diagnosis, it still could be a potential source of bias. Fourthly, other image parameters including the vertical pons hyperintensity which has been reported to be more sensitive than “hot cross bun” sign and “swallow-tail” sign were not evaluated (16, 20). Finally, the absence of a definite postmortem diagnosis increases the likelihood of misdiagnosis in our patients. Patients in the PD group included in the study had a relatively short disease duration (3.2 ± 2.8 years), and it cannot be said with great certainty that some of these patients will not turn out to have MSA-P several years later. Additional studies that investigate the association between DWI and pathological relations are needed.

In conclusion, we evaluated hypointense posterolateral putaminal signal in head DWI—the “black straight-line sign”. This sign had a favorable applicative value comparable to the hypointense putaminal posterolateral signal in SWI. The “black straight-line sign” may be added as a potential imaging marker for the diagnosis of MSA. It will be valuable for differentially diagnosing MSA, PD, and normal subjects in clinical practice.

## Data Availability Statement

The original contributions presented in the study are included in the article/supplementary material, further inquiries can be directed to the corresponding authors.

## Ethics Statement

The studies involving human participants were reviewed and approved by Institutional Review Board and Ethics Committee at Peking University First Hospital. The patients/participants provided their written informed consent to participate in this study. Written informed consent was obtained from the individual(s) for the publication of any potentially identifiable images or data included in this article.

## Author Contributions

YZhe and YS contributed to the concept and drafting and revision of the manuscript. XW, YJ, JC, WS, and HZ contributed to the collection of images and clinical data. ZW and YZhu contributed to revision of the manuscript. All authors have read and approved the final manuscript.

## Funding

The study was supported by Scientific Research Seed Fund of Peking University First Hospital (2018SF033). Funding bodies did not play a role in the collection, analysis, and interpretation of data. Funding bodies did not contribute to the writing of this manuscript.

## Conflict of Interest

The authors declare that the research was conducted in the absence of any commercial or financial relationships that could be construed as a potential conflict of interest.

## Publisher's Note

All claims expressed in this article are solely those of the authors and do not necessarily represent those of their affiliated organizations, or those of the publisher, the editors and the reviewers. Any product that may be evaluated in this article, or claim that may be made by its manufacturer, is not guaranteed or endorsed by the publisher.
